# Comparison of the antihypertensive efficacy of morning and bedtime dosing on reducing morning blood pressure surge: A protocol for systemic review and meta-analysis

**DOI:** 10.1097/MD.0000000000024127

**Published:** 2021-02-05

**Authors:** Peng Zhang, Mei-Ying Jin, Xu-Yu Song, Zhao Wang, Yue-Hua Jiang, Chuan-Hua Yang

**Affiliations:** a First Clinical Medical College, Shandong University of Traditional Chinese Medicine, Jinan 250355; b Yanzhou District Hospital of Traditional Chinese Medicine, Jining 272100; c Shandong College of Traditional Chinese Medicine; d Affiliated Hospital of Shandong University of Traditional Chinese Medicine, Jinan 250014, China.

**Keywords:** antihypertensive drugs, bedtime, hypertension, meta-analysis, morning blood pressure surge

## Abstract

**Background:**

It is well known that morning blood pressure surge increases the risk of myocardial events in the first several hours post-awakening. This meta-analysis was performed to compare the antihypertensive efficacy of morning and bedtime dosing on decreasing morning blood pressure surge.

**Methods:**

Articles in 4 databases about clinical trials of ingestion time of antihypertensive drugs were searched and performed a meta-analysis to evaluate the different effects on morning blood pressure and absolute blood pressure (BP) reduction from baseline of between bedtime administration (experimental group) and morning awaking administration (control group).

**Results:**

The aim of this study is to compare the antihypertensive efficacy of morning and bedtime dosing on decreasing morning blood pressure surge.

**Conclusions:**

The bedtime will provide evidence support for clinicians and patients for reducing morning blood pressure surge.

**Ethics and dissemination:**

This study does not require ethical approval.

## 1 Introduction

Hypertension is a global public health problem, causing many complications, especially cardiovascular events. The latest study (PURE) showed that hypertension was the largest risk factor and 22.3% of cardiovascular disease (CAD) cases and deaths were attributed to hypertension.^[1]^ Recent studies have shown that the morning blood pressure surge was a strong predictor of CAD. The morning blood pressure surge has a significantly increased risk of cardiovascular events, so effective control on the morning blood pressure is of substantial clinical importance for the management of hypertension. In the HONEST study, cardiovascular risk was higher in patients with morning blood pressure [BP] > 145 mm Hg and office BP < 130 mm Hg (hazard ratio [HR], 2.47; 95% confidence interval [CI], 1.20–5.08) than in patients with morning home BP < 125 mm Hg and office BP < 130 mm Hg.^[2]^ A cohort study showed that a 1-mm Hg increase in morning BP was associated with a 2.1% increased risk of cardiovascular death.^[3,4]^ Multiple prospective clinical trials indicate that improved normalization of asleep BP and 24 hours BP patterning – increase in sleep-time relative BP decline toward the more normal dipper profile – when administration with conventionally formulated single and combination hypertension medications at bedtime than upon awakening,^[5,6]^ without an increase in adverse effects.^[7]^ Compared with morning dosing, taking antihypertensives at bedtime nearly halves cardiovascular deaths, according to recent studies.^[5,8]^ In order to improve BP management, administration of antihypertensive drugs at bedtime has already been taken into account for the treatment strategy. Various researches focused on the influence of taking medicine before going to bed on 24 hours BP or cardiovascular events, and proposed that ingesting at least 1 antihypertensive medication at bedtime, compared with treatment with all medications upon awakening, gained a significant reduction in the 24-hours mean systolic BP (SBP)/diastolic BP (DBP) and the reduction was much more prominent during night time, decreasing the prevalence of non-dipping.^[9–11]^ However, no consensus has been reached on the morning surge BP-lowering effect of this strategy.

Therefore, we conducted this meta-analysis to assess the effects of bedtime administration of BP-lowering agents on the morning surge in BP monitoring (ambulatory blood pressure measurement [ABPM]) results.

## 2 Methods

### 2.1 Study registration

This NMA has been registered on the International Platform of Registered Systematic Review and Meta-analysis Protocols (INPLASY). The registration number is INPLASY2020110126.

### 2.2 Search strategy

This meta-analysis was conducted following (Preferred Reporting Items for Systematic Reviews and Meta-Analyses) PRISMA guidelines.^[12]^ The Literature search was performed in Pubmed, Embase, Cocharane, and ISI Web of Science without language restrictions, using the following search terms: Antihypertensive-drugs, Antihypertensive, Antihypertensive effect, Antihypertensive treatment, morning, awakening, blood pressure, Blood Pressure Monitoring, Ambulatory, bedtime, Morning blood pressure surge, Experimental trials, Treatment duration of antihypertensive drugs, Angiotensin-converting enzyme inhibitors, calcium channel blockers, beta-blockers, diuretics, angiotensin receptor blockers, and alpha-blockers, ABPM, SBP, DBP, Randomized controlled trial, Controlled clinical trial, Randomized, random allocation, without imposed language restrictions. The reference lists of all retrieved articles were also reviewed to identify additional articles missed by using these search terms. The specific search strategy of the PubMed database is shown in Table 1.

**Table 1:** Details of the search strategy for PubMed.

No.	Search item
#1	Hypertension [Mesh]
#2	(((Antihypertensive-drugs [Mesh]) OR (Antihypertensive [Mesh])) OR (Antihypertensive effect [Title/Abstract])) OR (Antihypertensive treatment [Title/Abstract])
#3	#1 OR #2
#4	Morning [Title/Abstract] OR awakening [Title/Abstract] OR blood pressure [Title/Abstract] OR Blood Pressure Monitoring [Title/Abstract]
#5	Morning blood pressure surge [Title/Abstract] OR ABPM [Title/Abstract] OR SBP[Title/Abstract] OR DBP [Title/Abstract]
#6	Ambulatory [Title/Abstract] OR bedtime [Title/Abstract]
#7	#4 OR #5 OR #6
#8	Experimental trials [Title/Abstract] OR Treatment duration of antihypertensive drugs [Title/Abstract]
#9	Calcium channel blockers [Title/Abstract] OR beta-blockers [Title/Abstract] OR diuretics [Title/Abstract] OR Angiotensin-converting enzyme inhibitors [Title/Abstract]
#10	angiotensin receptor blockers [Title/Abstract] OR alpha-blockers [Title/Abstract]
#11	#8 OR #9 OR #10
#12	(Randomized controlled trial) [Publication Type] OR (Controlled clinical trial [Publication Type])
#13	(Randomized [Title/Abstract]) OR (random allocation [Title/Abstract])
#14	#12 OR #13
#15	#7 AND #11 AND #14

### 2.3 Selection criteria

Studies were included if they met the following criteria:

1) Adult patients who satisfied diagnosis of hypertension using office measurements of blood pressure (SBP ≥ 140 mm Hg or DBP ≥ 90 mm Hg),^[13]^ including essential hypertension and secondary hypertension.
2) Experimental trials with at least 6 weeks’ treatment duration of antihypertensive drugs (angiotensin-converting enzyme inhibitors, calcium channel blockers, beta-blockers, diuretics, angiotensin receptor blockers, and alpha-blockers).
3) Intervention was defined as one or more antihypertensive drugs administered at bedtime (from 5:00 pm to 12:00 midnight), the control group was matched to the experimental group by drug and dose but with a morning regimen or in awaking time (from 6:00 am to 12:00 noon).
4) Outcomes: Morning SBP and morning DBP.
5) The pre- and post-treatment SBP and DBP of each patient were measured by ABPM, which is now the gold standard measurement and the most cost-effective strategy for diagnosing hypertension, evaluating true BP level.^[14]^ ABPM is recognized as a predictor of CV morbidity and mortality superior to office BP measurements.^[15]^

### 2.4 Data extraction

We extracted the data from each study included: the first author, year of publication, study country, sample size, patient characteristics, interventions (grouping, types of drugs, intervention duration), and study design.

### 2.5 Methodological assessment

We resolved any discrepancies by discussion and examined the obtained data carefully for accuracy, and assessed the methodological quality of the included studies independently using the risk of bias tools according to the Cochrane Handbook version 5.1.0.

### 2.6 Subgroup analysis

When there is heterogeneity between research results, we will conduct a comprehensive and systematic analysis of the reasons for heterogeneity and carry out hierarchical treatment according to different sources of heterogeneity. If it is due to the variation between studies, the intervention measures are consistent, and the research objects come from different populations. The following aspects will be used: age, course of the disease, gender.

## 3 Risk of bias

Risk of bias for each study was calculated in accordance with the Cochrane Handbook for Systematic Reviews of Intervention.^[16]^ Six criteria were applied as follows:

1) selection bias (random-sequence generation and allocation concealment);
2) performance bias (blinding of participants and personnel);
3) detection bias (blinding of outcome assessment);
4) attrition bias (incomplete outcome data);
5) reporting bias (selective reporting);
6) other bias.

There were 3 potential bias judgments:

1) low risk;
2) high risk;
3) unclear risk.

A study was rated as having an unclear risk when insufficient details were reported regarding the methods and outcome, the risk of bias was unknown, a metric was not relevant to the study, particularly for assessing blinding and incomplete outcome data, or when the outcome assessed by the metric had not been measured in the study.

## 4 Statistical analysis

All included studies were grouped according to the intervention regimen. The data from each included trial were analyzed using Review Manager (RevMan, Version 5.1, Copenhagen: The Nordic Cochrane Centre, The Cochrane Collaboration, 2011). The meta-analysis was performed using the generic inverse variance.

## 5 Statistical heterogeneity and sensitivity analysis

Statistical heterogeneity among studies was assessed using *I*^2^ and *Q* test statistic. Mild, moderate, and severe heterogeneity were defined by *I*^2^ values of 25%, 50%, and 75%, respectively. Sensitivity analysis was used to determine whether the included literature had a significant impact on the stability of the study.

## 6 Bias test

The funnel plot and Begger's test were used to assess the presence of publication bias.

## 7 Discussion

The incidence of cardiovascular events, such as myocardial infarction, sudden death, and stroke, is highest in the early hours after waking.^[17,18]^ The mechanisms involved in the morning increase in cardiovascular events have been unclear, but recent clinical studies have shown that an exaggerated morning BP surge is a plausible factor involved in the triggering of cardiovascular events, particularly in the case of stroke.^[18,19]^ 1 mm Hg morning BP increase was associated with a 2.1% increased risk of cardiovascular death.^[3,4]^ More and more evidences^[20–22]^ show that the morning surge of blood pressure is closely related to cardiovascular events, stroke, and renal impairment. And for medication, guidelines suggest the long-term antihypertensive drugs of 24-hour stable blood pressure can control the large fluctuation of morning blood pressure and reduce the rise of morning peak blood pressure caused by failure to take medicine on time or missing. It could be suggested to non-dipper type or anti-dipper type hypertension patients to take long-term antihypertensive drugs before going to bed. A study^[23]^ showed that the morning surge of BP, a risk factor for stroke, was significantly reduced only after bedtime administration of nifedipine. Bedtime in comparison to awakening-time ingestion of nifedipine was also associated with a reduction in the incidence of edema from 13% to 1%. Taking antihypertensive drugs before going to bed can improve the compliance of patients, and some commonly used drugs, such as statins, in the same way, are suggested to be taken before going to bed. Especially for many elderly people who live a lifestyle of get up early and doing morning exercises tend to be affected by morning blood pressure surge.

Still, it is controversial. Some opposing views are that the sharp decrease in blood pressure at night would bring adverse effects on organ blood supply. A study^[24]^ showed that although no obvious difference was found in adverse drug reactions between the 2 groups (patients were provided with a single pill containing amlodipine/atorvastatin (5/20 mg) to be taken each night at 10 pm*vs* patients were taking amlodipine (5 mg) and atorvastatin (20 mg) each morning at 7 am), compliance was much better in the single-pill group than in patients taking 2 medications separately. The latest research^[8]^ showed that taking antihypertensives at bedtime nearly halved cardiovascular deaths when compared with morning dosing.

The mechanism for these observed time administration of antihypertensive drugs differences remains unclear, but it has been generally agreed that the pharmacokinetics (PK) and pharmacodynamics (PD) of the medications occurring about to with concerning the 24 hours cyclic processes were involved in BP regulation.^[10,25]^ Circadian rhythms in gastric pH and emptying, gastrointestinal motility, biliary function and circulation, liver enzyme activity, and blood flow to the duodenum, kidney and other organs lead to PK and PD differences of antihypertensive medications due to different medication time.^[10,25]^ In particular, the circadian pattern of the glomerular filtration rate, with a maximum during the day and a minimum at night, played a significant role in PK.^[10,25,26]^ Antihypertensive drugs were eliminated more slowly overnight, potentially prolonging their duration of action when ingested at bedtime as compared to taken at awakening. The result of Hermida et al's study^[10]^ was consistent with the theory. The BP-lowering efficacy duration of spirapril was much longer when taken at bedtime (8 hours after ingestion) than in the morning (3 hours after ingestion). These studies indicated that antihypertensive drugs taken at bedtime demonstrated more effectively control on the morning surge of blood pressure. Moreover, the medications widely used in clinical conditions may have short half-life and duration of action and fail to provide a full 24 hours coverage, especially the morning surge of blood pressure, when taken in the morning.

## Author contributions

**Conceptualization:** Peng Zhang, Chuanhua Yang.

**Formal analysis:** Meiying Jin, Xuyu Song.

**Methodology:** Xuyu Song, Zhao Wang.

**Project administration:** Yuehua Jiang.

**Software:** Peng Zhang, Xuyu Song.

**Writing – original draft:** Peng Zhang.

**Writing – review & editing:** Chuanhua Yang.
